# Sensing Microbial RNA in the Cytosol

**DOI:** 10.3389/fimmu.2013.00468

**Published:** 2013-12-25

**Authors:** Nicolas Vabret, J. Magarian Blander

**Affiliations:** ^1^Department of Medicine, Immunology Institute, Icahn School of Medicine at Mount Sinai, New York, NY, USA; ^2^Tisch Cancer Institute, Icahn School of Medicine at Mount Sinai, New York, NY, USA

**Keywords:** pattern recognition receptors, RIG-I-like receptors, pathogen-associated molecular patterns, RNA helicases, cytosol, DExD/H-box helicases, innate immune escape

## Abstract

The innate immune system faces the difficult task of keeping a fine balance between sensitive detection of microbial presence and avoidance of autoimmunity. To this aim, key mechanisms of innate responses rely on isolation of pathogens in specialized subcellular compartments, or sensing of specific microbial patterns absent from the host. Efficient detection of foreign RNA in the cytosol requires an additional layer of complexity from the immune system. In this particular case, innate sensors should be able to distinguish self and non-self molecules that share several similar properties. In this review, we discuss this interplay between cytosolic pattern recognition receptors and the microbial RNA they detect. We describe how microbial RNAs gain access to the cytosol, which receptors they activate and counter-strategies developed by microorganisms to avoid this response.

## Introduction

When Janeway formulated the theory of pattern recognition in 1989, he proposed that host cells could sense microbial infection owing to receptors able to recognize invariant molecular structures defined as pathogen-associated molecular patterns (PAMPs). These patterns would be present in groups of pathogens, but absent in the host (1). Years later, Janeway and Medzhitov described the activity of the first mammalian member of the Toll-like receptor (TLR) family, Toll-like receptor 4 (2). TLRs comprise a family of transmembrane proteins able to recognize conserved microbial features and activate the immune response (3). Once activated, TLRs and others pattern recognition receptors (PRRs) initiate several intracellular pathways, including those mediated by nuclear factor-κB (NF-κB), mitogen-activated protein kinases (MAPKs), and interferon regulatory factors (IRFs). Another outcome of activation of distinct members of cytosolic PRRs is their oligomerization into multimeric cytosolic structures called inflammasomes, which activate the cysteine protease caspase-1, subsequently leading to the production of biologically active forms of pro-inflammatory cytokines (4).

Initially thought to detect exclusively microbial derived ligands, PRRs were later shown to recognize host derived danger signals, which are released in response to stress conditions such as cellular damage or tissue injury (3). Under normal physiological conditions, these ligands are not accessible to their respective PRRs and do not activate the immune system. Conversely, it was first suggested that self-DNA artificially introduced into the cytoplasm by transfection could activate NF-κB and the MAPK pathway (5). Evidence that any DNA, regardless of its origin, can engage innate immune receptors when localized outside of the nucleus was further confirmed by the identification of several endosomal and cytosolic DNA sensors [reviewed in Ref. (6)].

In contrast to cytosolic DNA, RNA sensing in the cytoplasm raises many questions on the mechanisms used by the innate system to specifically distinguish non-self-RNA from self-RNA. During infection, microbial RNAs share the cytosolic cellular compartment with several host RNA species, including messenger RNA (mRNA), transfer RNA (tRNA), ribosomal RNA (rRNA), microRNA, and other small regulatory RNAs. As a consequence, cytosolic sensors must display a high affinity for specific microbial features to avoid activation by host molecules that would otherwise elicit autoimmune responses. Despite this apparent challenge, efficient detection of foreign RNA in the cytosol is essential for innate immunity. During certain viral infections, RNA may be the only microbial PAMP produced throughout most of the replication cycle. Additionally, our laboratory previously showed that recognition of bacterial mRNA in the cytosol was critical to elicit a robust innate response against bacterial infection (7). Finally, cytosolic sensing of pathogen invasion by non-immune infected cells provides the very first steps of innate response against infection, before phagocytosis-competent immune cells are recruited to the site of infection.

In this review, we summarize the current understanding of cytosolic RNA sensing. We describe instances in which microbial RNAs gain access to the cytosol, the PRRs they activate, their corresponding ligands and strategies developed by microorganisms to conceal their RNAs.

## RNA Access to the Cytosol

**Figure 1:**
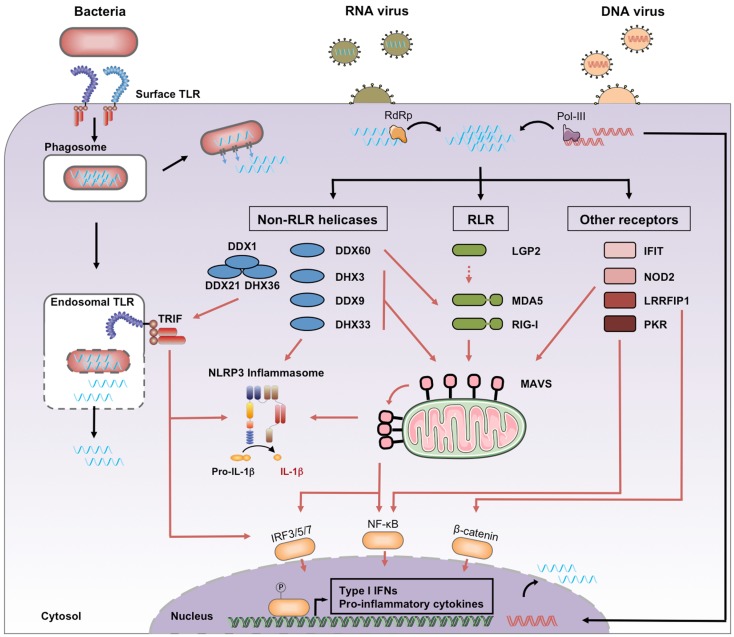
**Cytosolic recognition of microbial RNA**. Genomic RNA from RNA viruses access the cytosol immediately after the cell entry step of the replication cycle, where it may be amplified by viral RNA-dependent RNA polymerase (RdRp). Genomic DNA from DNA viruses is transcribed by viral or cellular RNA polymerase, including the cytosolic RNA polymerase III. Bacterial RNA can access the cytosol through the activity of auxiliary secretion systems or during passive leakage of phagosomal products. Once in the cytosol, microbial RNA binds different families of PRRs classified as RLRs, non-RLR helicases, and other receptors. Downstream signaling pathways include activation of MAVS, TRIF, and the NLRP3 inflammasome. Black arrows, RNA entry; red arrows, signaling pathways.

RNA entry into host cells generally takes place during the first steps of a microbial infection. We distinguish four processes leading to the presence of microbial RNA in the cytosol of eukaryotic cells, where it can engage host PRRs (Figure 1).

### Release of the viral genome and transcription of replication intermediates

A first process, observed during RNA virus infection, consists of viral genome release into the cytosol during the cell entry step of the replication cycle. Viruses can directly release their genome at the plasma membrane after binding to a receptor. Alternatively, they can be first internalized through endocytosis or macropinocytosis. Endocytosed virus particles will typically traffic through endosomal vesicles by actin-dependent and/or microtubule-dependent transport (8). Specific environmental triggers like endosomal pH acidification induce either fusion of enveloped virus with the endosome, or membrane penetration of viral proteins, allowing viral genetic material to be released into the cytoplasm (8). Alternatively, viruses can spread by direct cell–cell contact (9). Cell-to-cell transmission of viral material can activate cytoplasmic innate pathways, as exemplified with hepatitis C virus (10), lymphocytic choriomeningitis virus (11), or human immunodeficiency virus transmission (12).

Other viral RNA PAMPs can be produced during viral replication. David Baltimore has defined a classification of viruses based on the mechanism of mRNA production (13). Viruses are clustered in seven groups depending on their genomes (DNA, RNA), strandedness (single or double), sense or antisense, and method of replication. The type of RNA ligands produced during viral replication will depend on the type of viral genome and the strategy used to generate mRNA. RNA ligands can be generated by DNA viruses and retroviruses via genome transcription, or by synthesis of mRNA and replication intermediates by RNA-dependent RNA polymerases (RdRps) of RNA viruses (8).

### Phagosomal leakage of microbial ligands

It has been shown that ligands generated in phagolysosomes after phagocytosis of bacteria by immune cells can engage cytosolic innate immune receptors (14). Similarly, we showed that RNA from *Escherichia coli* could activate receptors in the cytosol after phagocytosis by macrophages (7). We demonstrated that phagosomes carrying *E. coli* exhibit intrinsic leakiness, suggesting a mechanism by which bacterial RNA, irrespective of the activity of virulence factors, can gain access to the cytoplasm (7).

### Active translocation of bacterial RNA to the cytoplasm

Alternatively, bacteria express secretion systems to translocate products outside of the bacterial cell wall. In the case of intracellular bacteria, auxiliary secretion systems like SecA2 in *Listeria monocytogenes* have been shown to actively translocate *Listeria* RNA into the cytoplasm, resulting in activation of cytosolic sensors (15, 16). Similarly, another study proved that cytosolic RNA sensors participate in the type 1 interferon (IFN-I) response to *Legionella pneumophila*. Although the authors did not demonstrate the translocation of *Legionella* RNA into the cytosol of infected cells, they discuss their data through a model where it would be the case (17). Future studies looking for additional secreted RNA will likely provide additional insights on their interaction with the innate immune system.

### Activity of RNA polymerase III

Two independent groups have demonstrated that cytoplasmic dsDNA triggers IFN-I production via RNA polymerase III, which transcribes DNA into 5’-triphosphate (5′-ppp) RNA, subsequently recognized by cytosolic RNA receptors (18, 19). This pathway has been involved in the sensing of DNA viruses, like Epstein–Barr virus, or intracellular bacteria, like *L. pneumophila* (18, 19). Although the RNA intermediate produced is not *sensu stricto* microbial, its generation is due to the activity of a microbial invader.

## Known Cytosolic RNA Sensors and Their Ligands

The best-studied cytosolic RNA sensors are the three members of RIG-I-like Receptors (RLRs), a subfamily of the DExD/H-box family of helicases. They consist of retinoic acid-inducible gene I (RIG-I), melanoma differentiation factor 5 (MDA5), and laboratory of genetics and physiology 2 (LGP2). They share a similar organization with three distinct domains: (i) a C-terminal repressor domain (RD) embedded within the C-terminal domain (CTD); (ii) a central ATPase containing DExD/H-box helicase domain able to bind RNA; and (iii) a N-terminal tandem CARD domain that mediates downstream signaling, and which is present in RIG-I and MDA5 but absent in LGP2. Upon activation by RNA ligands, RIG-I and MDA5 are subsequently recruited to the adaptor protein Mitochondrial Antiviral Signaling (MAVS) via a CARD–CARD interaction and lead to activation of NF-κB and IRFs (20–23). In contrast to TLR expression that is predominantly expressed in specialized immune cells such as macrophages and dendritic cells (DCs), RLRs are found in the cytosol of most cell types and are strongly induced in response to IFN-I (24, 25).

### Retinoic acid-inducible gene I

The RIG-I ligand has been characterized as an RNA molecule with two distinct features: (i) a 5′-ppp moiety (26, 27) and (ii) blunt-end base pair at the 5’-end (28, 29). Blunt-end base pairs can be found in double-stranded RNA (dsRNA) and secondary RNA structures such as hairpin or panhandle conformations (28, 29). Recent structural studies have contributed toward a better understanding of ligand binding and activation of RIG-I. Specificity of 5′-ppp binding is conferred by the CTD, and the helicase domain binds the double-stranded part of the RNA. RIG-I is normally held in an auto-repressed conformation, and ligand binding results in a conformational change, releasing the CARD domain which can subsequently initiate signaling by association with MAVS (30–32). Despite the increasing amount of high-resolution crystal data, the consensus definition of RIG-I ligand remains controversial. Other RIG-I ligands have been indeed described in the literature including long (33) or short dsRNA (34–36) lacking the 5′-ppp. However, thermodynamic analysis have shown that the full-length human RIG-I protein binds 5’-ppp dsRNA with 126-fold higher affinity than 5’-OH dsRNA, and dsRNA with a 361-fold higher affinity than short single stranded RNA (ssRNA) lacking secondary structure (37).

Many viral families display blunt-end base-paired RNA with a 5′-ppp, directly in their genomic RNA or in replication intermediates. Consistent with this notion, RIG-I has been shown to be involved in the recognition of many viruses, either antisense (−)ssRNA viruses (38, 39) or sense (+)ssRNA/dsRNA viruses (40, 41). Notably RIG-I can detect panhandle structures found in LaCrosse viral particles (39) or in influenza genomic RNA (28, 38). Sendai Virus (SeV) and other *Mononegavirales* produce defective interfering (DI) viral genomes containing panhandle structures that activate RIG-I in infected cells (42).

Retinoic acid-inducible gene I recognition has not been limited to RNA virus since RIG-I is involved in recognition of DNA viruses, such as Epstein–Barr virus or adenovirus through the RNA polymerase III pathway (18, 43, 44). Moreover, RIG-I is also able to detect bacterial infections. Bacterial mRNA are not capped and it has been estimated that approximately 40% of RNA oligonucleotides in *E. coli* have a 5′-ppp (45). Reports in the literature describe sensing of *L. monocytogenes* secreted RNA (15, 16) or purified *Legionella* (17) and *Helicobacter pylori* RNA (46) by RIG-I. Finally, RIG-I can also sense *Shigella flexneri* infection in macrophages through the RNA polymerase III pathway (47).

### Melanoma differentiation factor 5

Melanoma differentiation factor 5 ligand is less characterized than RIG-I. Using poly(I:C) as a synthetic dsRNA mimic, studies have shown that MDA5 binds long, but not short dsRNA (35, 40, 48). Structural analyses have demonstrated that MDA5 specifically recognizes the internal duplex structure of dsRNA and uses it as a platform to stack along dsRNA in a head-to-tail arrangement. This mechanism promotes stochastic assembly of the tandem CARD oligomers that activates the signaling adaptor MAVS (49).

Melanoma differentiation factor 5 detects infection by viral families known to produce long dsRNA structures during their replication cycle, including (+)ssRNA viruses like picornaviruses, dsRNA viruses like reoviruses, or DNA viruses like poxviruses (35, 50–53). In the case of (+)ssRNA virus infection, fluorescent imaging studies have confirmed that MDA5 recognizes preferentially the dsRNA generated during the replication of these viruses, but not the genomic ssRNA (54).

Prior to the structural study mentioned above, multiple observations raised the possibility that there may exist additional MDA5 ligands, different from the consensus long dsRNA. Thus, a study has shown that MDA5 cooperates with the ribonuclease RNase L to induce IFN-I in response to a viral mRNA from parainfluenza 5 virus (55). Interestingly, RNase L converts RNA into small RNA products, with shorter length than the current MDA5 ligand definition (56). Another work published the same year has shown that mRNA lacking 2’-O-methylation at their 5’ cap structure induces production of IFN-I through MDA5 activation (57). However, the data published, which focus on coronavirus infection, did not elucidate whether the absence of methylation was directly recognized by MDA5 or via another intermediate (57).

### MAVS mediates signaling downstream of RIG-I and MDA5

After binding to their specific ligands, both RIG-I and MDA5 activate MAVS to trigger a common signaling pathway. The majority of MAVS is localized on the mitochondrial membrane and its engagement by RLRs causes a conformational change that propagates to adjacent un-activated MAVS in a prion-like behavior (58). The formation of these very large MAVS aggregates results in a large-scale amplification of the signaling cascade. This cascade involves the recruitment of cytosolic adaptor molecules, followed by the activation of the canonical IKKs, IKK-α, IKK-β, and IKK-γ, the MAPK and the non-canonical IKK-related kinase, TBK1 and IKK-i/ε. Ultimately, specific transcription factors, such as IRF3, NF-κB, and depending on the cell type IRF5 and IRF7, are translocated to the nucleus where they promote the expression of IFN-I genes and pro-inflammatory cytokines [reviewed in Ref. (59)].

Finally, MAVS has been recently shown to interact with NOD-like receptor family, pyrin domain containing 3 (NLRP3) and promote its recruitment to the mitochondria. The authors emphasize the central role of MAVS in innate immune signaling events by showing its importance in the functioning of NLRP3 inflammasome and the production of IL-1β (60). Of note, MAVS independent activation of the NLRP3 inflammasome by RIG-I has also been reported (61, 62).

### Laboratory of genetics and physiology 2

The third member of RLRs, LGP2, is able to bind dsRNA (63, 64), however, its role in immune activation is poorly understood. LGP2 was proposed to be a modulator of RLR signaling. Studies showed that LGP2 was required for RIG-I and MDA5 activity, in particular during picornaviral infection (65–67). Another work proposed that LGP2 would inhibit RIG-I through competition with its ligand (64). It is however unclear whether LGP2 binds microbial RNA in an infectious context, and what specific features of the RNA it would recognize. Further studies will be required to clarify the precise role of LGP2.

### Non-RLR helicases

Apart from RLR, several recent studies have highlighted the importance of other DExD/H-box helicases in microbial RNA sensing. RNA helicases of the DEAD box family are involved in various different steps of RNA metabolism [reviewed in Ref. (68)]. They share eight conserved motifs that are involved in ATP binding, ATP hydrolysis, nucleic acid binding, and RNA unwinding activity. Additionally, most DExD/H-box helicases contain auxiliary N- and C-terminal domains that confer on them functional specificities, such as an ability to induce downstream signaling or to bind specific RNA targets (69).

### DDX3

DDX3 (DDX3X) can bind poly(I:C) or vesicular stomatitis virus (VSV) RNA and was shown to enhance the IFN-I response to VSV infection by interaction with the RLR-MAVS complex. Overexpression assays suggest that DDX3 precipitates with RIG-I and MDA5 (70). Since DDX3 is easily detected in resting cells, the authors propose a sentinel role for this helicase, the activity of which would be required during the initial steps of viral infection. Another study showed that upon SeV infection, DDX3 interacts with IKKε, an essential component of the IRF3 signaling pathway, increasing the induction of the IFN-β promoter (71). Moreover, DDX3 is targeted by vaccinia virus protein K7 (71), an inhibitor of IFN-β production, and by HCV core protein, which can disrupt its interaction with MAVS (72). These observations highlight the importance of DDX3 in efficient viral sensing.

### DHX9

Using overexpression and knock-down experiments, DHX9 was shown to be required for the production of IFN-I and pro-inflammatory cytokines in response to poly(I:C), influenza virus, and reovirus by a murine splenic DC line and bone-marrow derived DCs. DHX9 can bind dsRNA via its dsRNA-binding motif and interact with MAVS through both its helicase C-terminal domain and HA2-DUF (73).

### DDX1, DDX21, and DHX36

Myeloid DCs have also been shown to express a complex composed of DDX1, DDX21, and DHX36 that triggers an antiviral program in response to poly(I:C), in a pathway dependent of the adapter molecule TIR-domain containing adapter-inducing interferon-β (TRIF). DDX1 binds to poly(I:C) via its helicase A domain, while DHX36 and DDX21 bind the TIR domain of TRIF via their HA2-DUF and PRK domains, respectively. This complex seems to be required for the innate response against influenza or reovirus infection (74). Notably, a separate study also characterized DHX36 and DHX9 as a sensor for the dsDNA species CpG-A and -B, respectively. In this case, both DHX36 and DHX9 activate the cytosolic adapter protein myeloid differentiation primary response gene 88 (MyD88) by binding to its TIR domain (75).

### DHX33

Another recent study by Yong-Jun Liu’s group identified another helicase, DHX33, as a cytosolic RNA receptor able to activate the NLRP3 inflammasome (76). DHX33 is involved in inflammasome activation after sensing cytosolic RNA such as poly(I:C) or reoviral RNA when directly delivered by lipofection to the cytoplasm of a macrophage cell line or human monocyte-derived macrophages. Additional experiments suggested that DHX33 could also possibly be involved in detection of cytosolic bacterial RNA. The authors showed that DHX33 can bind to dsRNA through its helicase C domain and to NLPR3 through its DEAD domain (76). A few months later, another study performed on myeloid DCs confirmed the role of DHX33 in the sensing of cytosolic poly(I:C) and reoviral RNA. Surprisingly, in this case, poly(I:C)-induced activation of MAPK, NF-κB, and IRF3 was mediated by MAVS, which binds the helicase C domain of DHX33 (77).

### DDX60

DDX60 has also been shown to enhance the IFN-I response to RNA and DNA stimulation through formation of complexes with RIG-I, MDA5, and LGP2 but not with MAVS. This complex formation has been deciphered with overexpression assays in the case of MDA5 and LGP2, and with endogenous RIG-I during VSV infection. DDX60 expression is induced by viral infection and its helicase domain can bind ds- or ss-VSV RNA generated *in vitro*, independently of the 5′-ppp (78). Interestingly, DDX60 can also bind dsDNA, and was shown to play role in IFN-I expression after infection with Herpes Simplex Virus-1, a DNA virus. This ability to bind both dsRNA and DNA raises the question of the feature DDX60 recognizes. It should be finally noted that the role of DDX60 in the IFN-I pathway has been questioned (79).

## Other RNA Receptors

Several other cytoplasmic receptors have been shown to play a role in microbial RNA recognition. This is the case for the cytoplasmic protein kinase R (PKR), which is important for antiviral activity. PKR is activated by dsRNA from viruses and is a component of MAPK and NF-κB signaling pathways [reviewed in Ref. (80)]. Activation of PKR can also be mediated by short 5′-ppp RNAs containing limited secondary structures (81).

Proteins from the Interferon-induced protein with tetratricopeptide repeats (IFITs) family, such as IFIT1 and 5, bind 5′-ppp of viral RNA (82). Using short *in vitro* transcribed oligonucleotides, crystal structure studies have demonstrated that IFIT proteins contain a positively charged cavity designed to engage, without any particular sequence specificity, ssRNA with a 5′-ppp end. Contrary to RIG-I, IFIT proteins cannot bind blunt-ended 5′-ppp dsRNA, and owing to the limitations imposed by their RNA-binding pockets, IFIT1 and IFIT5 require 5’-overhangs of at least 5 or 3 nt, respectively (83).

Using a 2′-*O*-methyltransferase mutant of Japanese encephalitis virus, another study showed that IFIT1 preferentially binds to 5′ capped 2′-*O*-unmethylated mRNA (84), confirming previous findings showing that 2′-O-methylation of viral mRNA caps promotes IFIT1 evasion (85, 86). The mechanism of IFIT1 antiviral action is not completely understood, and it has been proposed that IFIT might sequester viral RNAs (82) or inhibit viral mRNA translation (84). The crystal structure of IFIT2 (known as ISG54) was also described. IFIT2 specifically binds adenylate uridylate (AU)-rich RNAs *in vitro*, independently of the presence of a 5′-ppp (87). The authors showed that RNA-binding capacity of this protein mediates its antiviral properties, using a model of HEK293T cells infected by Newcastle disease virus or SeV (87).

Nucleotide-binding oligomerization domain containing protein 2 (NOD2) is a member of the NOD1/Apaf-1 family and encodes a protein with two CARD domains and six leucine-rich repeats (LRRs). NOD2 is primarily known for its ability to recognize bacterial peptidoglycan, but it also plays a role in the antiviral response. NOD2 has been shown to activate MAVS after stimulation with viral ssRNA or human respiratory syncytial virus infection (88). NLRP3 is involved in cytosolic RNA sensing. Caspase-1 cleavage triggered by influenza virus, SeV, or bacterial mRNA is dependent on NLRP3 inflammasome activation (7, 89, 90). However, direct binding of NOD2 or NLRP3 to microbial RNA has not been established.

Leucine-rich repeat flightless-interacting protein 1 (LRRFIP1) contributes to the production of IFN-β induced by VSV and *L. monocytogenes* in macrophages (91). Mostly located in the cytosol, LRRFIP1 can also be found in RNA-containing lysosomes (92). LRRFIP1 can bind both dsRNA and dsDNA and subsequently induce IFN-I expression through β-catenin phosphorylation. Activated β-catenin is translocated to the nucleus and increases IFN-β expression by binding to the C-terminal domain of the transcription factor IRF3 and promoting the recruitment of the acetyltransferase p300 to the *Ifnb1* promoter.

## Immunostimulatory Features and Other Putative RNA PAMPs

Several other microbial RNA features have been suspected or proposed to act as potential signals for cytosolic sensing, suggesting the existence of receptors detecting these characteristics. A computational analysis identified CpG motifs in an AU-rich RNA as an immunostimulatory feature. This sequence motif is underrepresented in both ssRNA viruses and host innate immune gene mRNA, and its frequency in influenza virus genomes has decreased throughout evolution (93). Since this evolutionary pressure seems to also be applied on host mRNA, the implication of a cytosolic receptor is possible, although experimental studies identified endosomal TLR7 as a potential PRR (94). Another study identified the nucleotide bias of A-rich HIV-1 genome as a strong inducer of IFN-I and potent mediator of lentiviral pathogenicity. The authors showed that the ability of RNA sequences derived from the HIV-1 genome to induce an interferon response correlated with their nucleotide bias and that codon-optimized sequences lost their stimulatory activity (95). The experimental procedure used in this study consisted of direct delivery via lipofection of *in vitro* transcribed RNA sequences into the cytosol of a reporter cell line, suggesting a potential role for a cytoplasmic RNA sensor (95). Recently, our group identified bacterial mRNAs as an activator of the NLRP3 inflammasome. Polyadenylation of these RNAs abrogated their immunostimulatory activities, suggesting that features at the 3′ end of mRNA, rather than the 5′ end, could engage cytoplasmic cellular sensors (7).

Philip Bevilacqua’s group has shown that different nucleoside modifications on RNA, such as base or sugar internal modifications, suppress their intrinsic ability to activate immune sensors, notably PKR. The authors propose that self-RNA editing could be a mechanism used by the innate immune system to discriminate self-transcripts from “unmodified” microbial RNAs (96, 97). Conversely, microbial RNA editing by cellular deaminase enzymes such as dsRNA-specific adenosine deaminase (ADAR) have been shown to enhance its recognition by cytosolic sensors (98).

Other host transcript specificities, like association to cellular components that prevent PRR binding, or specific tertiary structure such as the eukaryotic mRNA closed loop conformation (99), could be determinants for the differentiation of host mRNAs from microbial RNAs. Identification of receptors able to recognize such features are lacking so far.

## Microbial Escape Strategies

Infectious microorganisms have developed several strategies to evade cytosolic sensing. One of these strategies, which we only mention briefly here, is the direct targeting by microbial proteins of host PRRs and molecules involved in downstream signaling pathways. Thus, many pathogens code for proteins that lower cellular levels of PRRs and signaling molecules or directly disrupt their antimicrobial activities [reviewed in Ref. (79, 100)]. Other strategies are discussed below.

## RNA Editing

Some (−)ssRNA viruses edit the 5′-ppp moieties in their genomes as well as replication intermediates into 5′ mono-phosphates to avoid recognition by RLRs (101). Arenaviruses produce RNA panhandle structures with a 5′-ppp containing a GTP overhanging nucleotide. This viral structure is suggested to act as a RIG-I ligand decoy, by trapping RIG-I but not activating it (102). We are beginning to understand how eukaryotic cells use nucleoside modifications in order to protect self-RNAs from innate sensing. For example, higher eukaryotes have acquired the ability to 2′-*O*-methylate their mRNAs, allowing cellular receptors to distinguish self from unmethylated non-self mRNA through specific types of antiviral sensors such as MDA5 and IFITs (57, 85). Consistent with the red queen hypothesis (103), which postulates that parasites have to constantly evolve in order to adapt to their host species, the same immune escape strategy has been mimicked by several pathogens, like flaviviruses (84, 86). Similarly, 2′-O-methylation of G18 (Gm18) on bacterial tRNA suppresses activation of the immune response in plasmacytoid DCs (104, 105).

## Compartmentalization in the Cytoplasm

Flaviviruses and other viruses are also known to induce cellular membrane reorganization that allows them to replicate in subcellular compartments, creating new replication-dependent organelles (106). Thus, tick-borne encephalitis virus or Japanese encephalitis virus have been shown to rearrange endoplasmic reticulum membranes to provide a compartment where viral dsRNA is concealed from PRR recognition. This hijacking of internal cell membrane induces a delayed cytosolic exposure of viral RNA to innate receptors and accordingly, IFN-I responses are only measured late in the replication cycle (107–109).

## Protecting or Degrading Ligands

The NS1 protein from influenza virus can prevent RNA sensing through the formation of a chain of NS1 molecules along the influenza dsRNA backbone (110). Picornaviruses mask their 5′-ppp with a viral encoded protein, VPg, which functions as a 5′ cap and as a primer during RNA synthesis. Interestingly, studies have shown that VPg could be used to evade RIG-I recognition (111). Similarly, Ebola virus VP35 assembles into dimmers to cap the ends of viral dsRNA and hide the specific RIG-I recognition site (112). While one VP35 monomer binds the terminus and backbone of dsRNA, the other VP35 monomer binds only the phosphate backbone of the dsRNA, displaying a unique mode of dsRNA concealing from PRR (112). Another hemorrhagic fever virus, Lassa fever virus, uses the 3’–5’ exonuclease activity of its nucleoprotein (NP) to degrade stimulatory dsRNA (113). This activity seems to be shared by other arenaviruses (114). Finally, the protein C from human parainfluenza virus type 1 (HPIV1), a paramyxoviridae, has been shown to limit the accumulation of dsRNA. Cell infection by a virus mutant defective for the C protein displays higher accumulation of several viral RNAs, including viral genome, antigenome, and mRNA, eventually leading to the accumulation of dsRNA. Thus, by limiting intracytosolic quantities of viral dsRNA, the C protein of HPIV1 avoids dsRNA triggering of MDA5 and PKR in infected cells (115).

## Concluding Remarks

The multiplicity of PRR pathways is an essential determinant of the immune system’s ability to sense with precision the level of microbial threat and to respond accordingly (4). However, as far as cytosolic RNA sensors are concerned, it is striking to observe the contrast between the high number of PRRs that have been isolated and the similarities of the PAMPs they recognize (Table 1). While 5′-ppp and dsRNA are undoubtedly powerful triggers of the innate immunity, they cannot account for the diversity of responses that the organism is able to elicit against a wide range of pathogens. Our understanding of how the immune system distinguishes between foreign and self-nucleic acids will continue to improve over time. This will help us better define the precise role played by cytosolic RNA sensors in the global immune response against pathogens.

**Table 1:** **Cytosolic RNA sensors and their ligands**.

RNA sensor	Proposed RNA ligand	Families of reported recognized pathogens
RIG-I	5′-ppp with blunt-end base pairing ssRNA; dsRNA	*(−)ssRNA* viruses, *(*+)*ssRNA* viruses, *dsRNA* viruses, bacteria. DNA viruses, and bacterial DNA through polymerase III pathway
MDA5	Long dsRNA	*(−)ssRNA* viruses, *(*+)*ssRNA* viruses, *dsRNA* viruses, *DNA* viruses
LGP2	dsRNA	*(*+)*ssRNA* viruses
DDX3	Viral RNA; poly(I:C)	*(−)ssRNA* viruses
DHX9	Viral RNA; poly(I:C)	*(−)ssRNA* viruses, *dsRNA* viruses
DDX1, DDX21, and DHX36	Viral RNA; poly(I:C)	*(−)ssRNA* viruses, *dsRNA* viruses
DHX33	Viral RNA; poly(I:C)	*dsRNA* viruses, bacteria
DDX60	*In vitro* transcribed ssRNA and dsRNA	*(−)ssRNA* viruses, *DNA* viruses
PKR	dsRNA; short 5′-ppp RNA	(−)*ssRNA* viruses, *(*+)*ssRNA* viruses, *dsRNA* viruses, *DNA* viruses
IFIT1 and IFIT5	5′-ppp ssRNA; 5′capped 2′-*O*-unmethylated RNA	*(−)ssRNA* viruses
NOD2	Viral ssRNA	*(−)ssRNA* viruses
NLRP3	dsRNA, bacterial RNA	*(−)ssRNA* viruses
LRRFIP1	dsRNA	*(−)ssRNA* viruses, bacteria

## Conflict of Interest Statement

The authors declare that the research was conducted in the absence of any commercial or financial relationships that could be construed as a potential conflict of interest.
